# Genomic and GWAS-Based Insights into Antimicrobial Resistance in *Shewanella algae* Isolated from *Penaeus monodon*

**DOI:** 10.3390/antibiotics15040405

**Published:** 2026-04-16

**Authors:** Ponsit Sathapondecha, Wichai Pornthanakasem, Timpika Thepsuwan, Pacharaporn Angthong, Wiyada Chumpol, Kamonwan Lunha, Suganya Yongkiettrakul, Wanilada Rungrassamee

**Affiliations:** 1 Division of Biological Science, Faculty of Science, Prince of Songkla University, Hat Yai 90110, Songkhla, Thailand; ponsit.s@psu.ac.th; 2 National Center for Genetic Engineering and Biotechnology, National Science and Technology Development Agency, 111 Thailand Science Park, Phahonyothin Road, Khlong Luang 12120, Pathum Thani, Thailand; wichai.por@biotec.or.th (W.P.); pacharaporn.ang@biotec.or.th (P.A.); wiyada.chu@ncr.nstda.or.th (W.C.); kamonwan.lun@ncr.nstda.or.th (K.L.); suganya.yon@biotec.or.th (S.Y.)

**Keywords:** *Shewanella algae*, *Penaeus monodon*, colistin resistance, *lptA*, genomic surveillance, One Health

## Abstract

**Background/Objectives:** The emergence of antimicrobial-resistant (AMR) pathogens in aquaculture ecosystems poses a significant risk to both food security and human health. *Shewanella* species are recognized as significant AMR reservoirs, yet their prevalence and resistance mechanisms within a shrimp-related ecosystem remain poorly characterized. This study aimed to perform a genotypic and phenotypic characterization of *S. algae* VK101, isolated from wild-caught black tiger shrimp (*Penaeus monodon*) broodstock. **Methods:** A complete 5.21 Mb genome was generated using a hybrid Illumina and Oxford Nanopore sequencing approach. Antimicrobial susceptibility was evaluated for 21 antibiotics via Minimum Inhibitory Concentration (MIC) testing. Comparative pangenomics and genome-wide association studies (GWAS) across 125 *S. algae* genomes were conducted to identify novel resistance determinants. **Results:** MIC analysis revealed that VK101 was resistant to ampicillin (>16 µg/mL) and colistin (8 µg/mL), while showing intermediate susceptibility to imipenem and ciprofloxacin. In silico analysis identified 205 antimicrobial resistance genes (ARGs), including a perfect hit for the fluoroquinolone resistance gene *qnrA3*. Notably, no *mcr* genes were detected. Although VK101 exhibited moderate resistance (8 µg/mL), GWAS across the broader *S. algae* population linked a specific *lptA* mutation (K140N) to high-level resistance (64 µg/mL). Other GWAS-identified genes (e.g., *czcA*, *ampC*, and *oprM*) likely represent indirect associations driven by genetic linkage or clade-specific markers rather than direct causal factors. **Conclusions:** These findings highlighted the presence of multidrug-resistant *S. algae* in wild-caught *P. monodon* broodstock, reflecting the occurrence of antimicrobial resistance in aquatic environments. Colistin resistance in these isolates was primarily mediated by chromosomal variants rather than mobile *mcr* elements, indicating the need for integrated genomic surveillance within the aquaculture value chain.

## 1. Introduction

Antimicrobial resistance (AMR), particularly bacterial AMR, has become a crucial global public health threat, compromising the efficacy of current therapeutic interventions and preventative measures against infectious diseases [1]. Bacterial AMR was associated with an estimated 5 million deaths in 2019, with this number predicted to increase substantially by 2050. Consequently, urgent action is needed to mitigate the dissemination of resistant determinants [2]. This challenge is particularly pronounced among Gram-negative species [3]. Due to increasingly limited treatment options, the World Health Organization (WHO) has designated many of these, including *Escherichia coli*, *Staphylococcus aureus*, *Klebsiella pneumoniae*, *Streptococcus pneumoniae*, *Acinetobacter baumannii*, and *Pseudomonas aeruginosa*, as high-priority pathogens [4]. Under the One Health framework, human health is intrinsically linked to animal health and the shared environment. Within this paradigm, environmental reservoirs function as critical sources of resistant genetic determinants and opportunistic pathogens, where resistance is driven by antimicrobial selection pressure, such as the systemic overuse of antimicrobials, natural adaptive mutations, and rapid environmental spread facilitated by horizontal gene transfer [5]. Moreover, both climate change and antibiotic exposure can alter microbial ecosystems, resulting in the induction of resistance mechanisms [6]. Importantly, multidrug resistance was detected, particularly to β-lactams, sulfonamides, tetracyclines, and fluoroquinolones, among pathogenic bacteria [7].

Within global animal production, aquaculture served as a critical interface for the dissemination of AMR determinants between environmental reservoirs and clinical settings [8]. This dissemination is evident in the cultivation of the black tiger shrimp (*Penaeus monodon*), an economically important species in Southeast Asia and Australia [9]. While the industry aims to shift toward domesticated broodstock to enhance biosecurity, a significant portion of production remains dependent on wild-caught individuals [10,11]. These wild populations harbor diverse and complex microbial communities, frequently dominated by the genera *Vibrio* and *Photobacterium* [12], which could be significant reservoirs for diverse resistance genes [13,14]. Consequently, the introduction of wild-caught broodstock into controlled hatchery systems may result in a management challenge, potentially integrating naturally occurring AMR bacteria into the production value chain.

Much of the research on AMR in aquaculture have focused extensively on the genus *Vibrio*, as these dominant marine bacteria are frequently associated with both host pathogenicity and the carriage of mobile antibiotic resistance genes (ARGs) [15,16]. While these studies have led to fundamental insights into the dissemination of resistance and the development of targeted mitigation strategies to effectively control *Vibrio* within farming environments [17], other ubiquitous and ecological bacteria genera remain under characterized. Specifically, members of the genus *Shewanella* have been reported as potential reservoirs for ARGs [8,18,19,20]. While considered ubiquitous environmental bacteria, several species, including *S. algae*, *S. putrefaciens*, and *S. xiamensis*, are now identified as emerging opportunistic pathogens in human clinical cases, associated with bacteremia and severe soft-tissue infections [21,22,23,24]. Despite their clinical relevance and ability to live on across diverse ecological niches, the characterization of *Shewanella* isolates originating from shrimp-related ecosystems remains limited. The transition toward sustainable, non-antibiotic disease management strategies, such as probiotics, vaccination, and enhanced biosecurity, is essential for the future of responsible aquaculture [25,26]. However, the successful implementation of these alternative methods requires a comprehensive genomic understanding of the baseline environmental AMR. Identifying specific chromosomal and mobile genetic determinants of resistance in under-studied genera like *Shewanella* is therefore important for designing interventions that effectively reduce and mitigate risks of AMR dissemination.

In this study, we reported a high-resolution genotypic and phenotypic characterization of *S. algae* VK101, isolated from the intestinal microbiome of a wild-caught *P. monodon* broodstock. By utilizing an integrated sequencing approach of short- and long-read platforms to resolve a complete 5.21 Mb circular genome, we established a definitive antimicrobial susceptibility profile against 21 antibiotics and identified the underlying genetic determinants of resistance. Our comparative analysis across a dataset of 125 *S. algae* genomes revealed that colistin resistance in these environmental isolates was likely driven by stable chromosomal variants rather than mobile *mcr* elements. Particularly, species-wide comparative analysis identified a K140N mutation in the lipopolysaccharide transport protein gene (*lptA*) in highly resistant isolates, suggesting a structurally based resistance mechanism that may have been overlooked by traditional molecular screening methods. Elucidating these pathways is essential for developing the rigorous genomic surveillance and comprehensive biosecurity protocols required to safeguard the aquaculture value chain and public health.

## 2. Results

### 2.1. Whole Bacterial Genome Annotation and Functional Prediction

**Figure 1:**
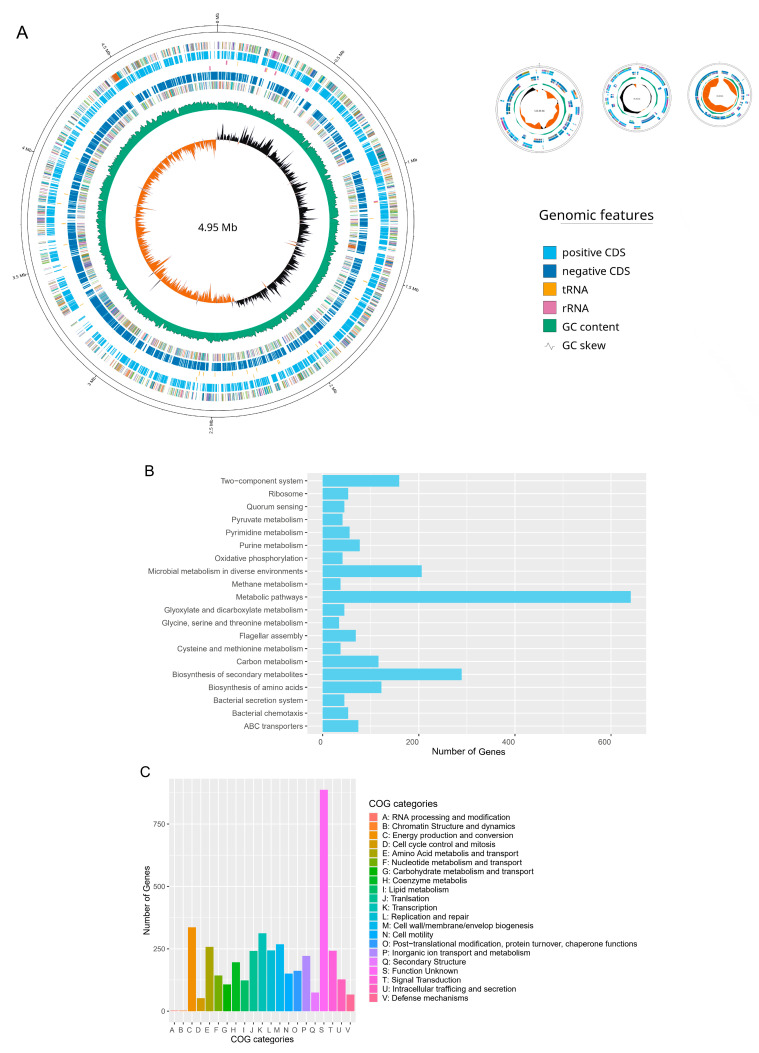
Complete genome architecture and functional landscape of *Shewanella algae* VK101. (**A**) Circular representation of the 5.21 Mb *S. algae* VK101 genome, (**B**) distribution of genes across KEGG metabolic pathways, and (**C**) COG functional classification of the VK101 proteome.

The complete genome of *S. algae* VK101 was successfully constructed using a hybrid assembly strategy that integrated 8,305,448 high-accuracy short reads with 305,874 long reads. The final assembly resulted in a genome size of 5.21 Mb, characterized by four complete circular contigs (Table 1 and Figure 1). The high-resolution assembly featured a dominant 4.95 Mb chromosome, and an average GC content of 52.6%, while the other three smaller contigs were 123.44 kb, 76.36 kb, and 66.66 kb, respectively. Genomic annotation identified 4620 coding sequences (CDS), 103 tRNAs, and 25 rRNAs, resulting in a coding ratio of 87.2% (Table 1 and Figure 1A). Functional annotation of the *S. algae* VK101 genome by KEGG pathway analysis showed that most genes were involved in metabolisms (Figure 1B). While metabolic pathways dominated the functional profile, a significant number of genes were related to the biosynthesis of secondary metabolites, microbial metabolism in diverse environments, and two-component system. Furthermore, classification through Clusters of Orthologous Groups (COG) showed that while most genes were classified under unknown function, the most prevalent categories were energy production and conversion, transcription, and cell wall/membrane/envelope biogenesis (Figure 1C). Conversely, genes associated with RNA processing and modification and chromatin structure and dynamics were infrequently represented in the VK101 genome (Figure 1C).

### 2.2. Phenotypic Susceptibility and Antimicrobial Profiling

The MIC values were 2 μg/mL of imipenem and less than or equal to 0.5 μg/mL of meropenem, ertapenem, and doripenem (Table 2). Moreover, the result indicated that the MIC values for ciprofloxacin and levofloxacin of the fluoroquinolone class were 0.5 and 0.25 μg/mL, respectively. The observation was consistent with our *in silico* analysis, and the MIC value of colistin was obtained at 8 μg/mL as the highest of the concentration range in the antibiotic plate, suggesting potential resistance to this antibiotic.

The VK101 isolate was susceptible to most beta-lactam/beta-lactamase inhibitor combinations, including ampicillin/sulbactam (8/4 µg/mL) and piperacillin/tazobactam (≤8/4 µg/mL). It also showed susceptibility to cephalosporins: cefoxitin (8 µg/mL), cefuroxime ≤8 µg/mL), cefotaxime (≤1 µg/mL), ceftazidime (≤1 µg/mL), ceftriaxone (≤0.5 µg/mL), and cefepime (≤1 µg/mL). Among carbapenems, VK101 remained susceptible to meropenem (≤0.5 µg/mL), ertapenem (≤0.5 µg/mL), and doripenem (≤0.5 µg/mL). Additional agents with full activity included fluoroquinolones (levofloxacin, 0.25 µg/mL), aminoglycosides (amikacin, ≤8 µg/mL; gentamicin, ≤2 µg/mL; netilmicin, ≤8 µg/mL), and trimethoprim/sulfamethoxazole (≤1/19 µg/mL). In contrast, VK101 exhibited intermediate susceptibility to amoxicillin/clavulanic acid (16/8 µg/mL), imipenem (2 µg/mL), and ciprofloxacin (0.5 µg/mL). Notably, the isolate was resistant to ampicillin (>16 µg/mL) and colistin (8 µg/mL) (Table 2).

### 2.3. Targeted Resistome Profiling in S. algae VK101

**Figure 2:**
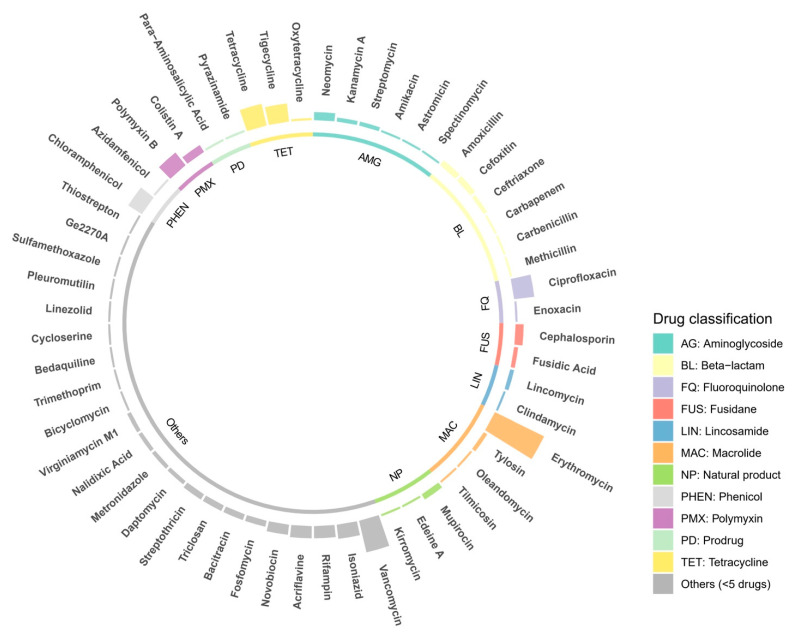
Circular distribution of the environmental resistome in *S. algae* VK101. Comparative circular plot identifying the diverse classes of antimicrobial resistance genes (ARGs) detected in the VK101 genome. Bar heights represent the relative abundance or match confidence for specific resistance determinants, including those for fluoroquinolones, beta-lactams, and polymyxins.

The complete genome of *S. algae* VK101 was performed in silico analysis to map its intrinsic genes and ARGs. Our screening identified a total of 205 ARGs, encompassing 420 loci, distributed across the chromosome and smaller contigs of the genome (Supplementary Materials, Table S1). The functional mapping of these determinants showed a diverse resistome profile (Figure 2), with significant hits identified for major compound classes such as fluoroquinolones, beta-lactams, and polymyxins.

Among these determinants, the quinolone resistance protein, QnrA3, was identified as a perfect hit (100% identity), providing a genetic basis for the intermediate susceptibility observed toward ciprofloxacin and levofloxacin (Table 3). Furthermore, additional hits were characterized including OXA-964 (98.96% identity), *rsmA* (86.67%), and *E. coli* EF-Tu mutants (88.3%), which collectively contributed to the isolate’s multidrug-resistant profile (Table 2). Interestingly, our genomic analysis did not identify *mcr*, the key ARG related to colistin resistance.

### 2.4. Comparative Pangenomic Analysis of S. algae

**Figure 3:**
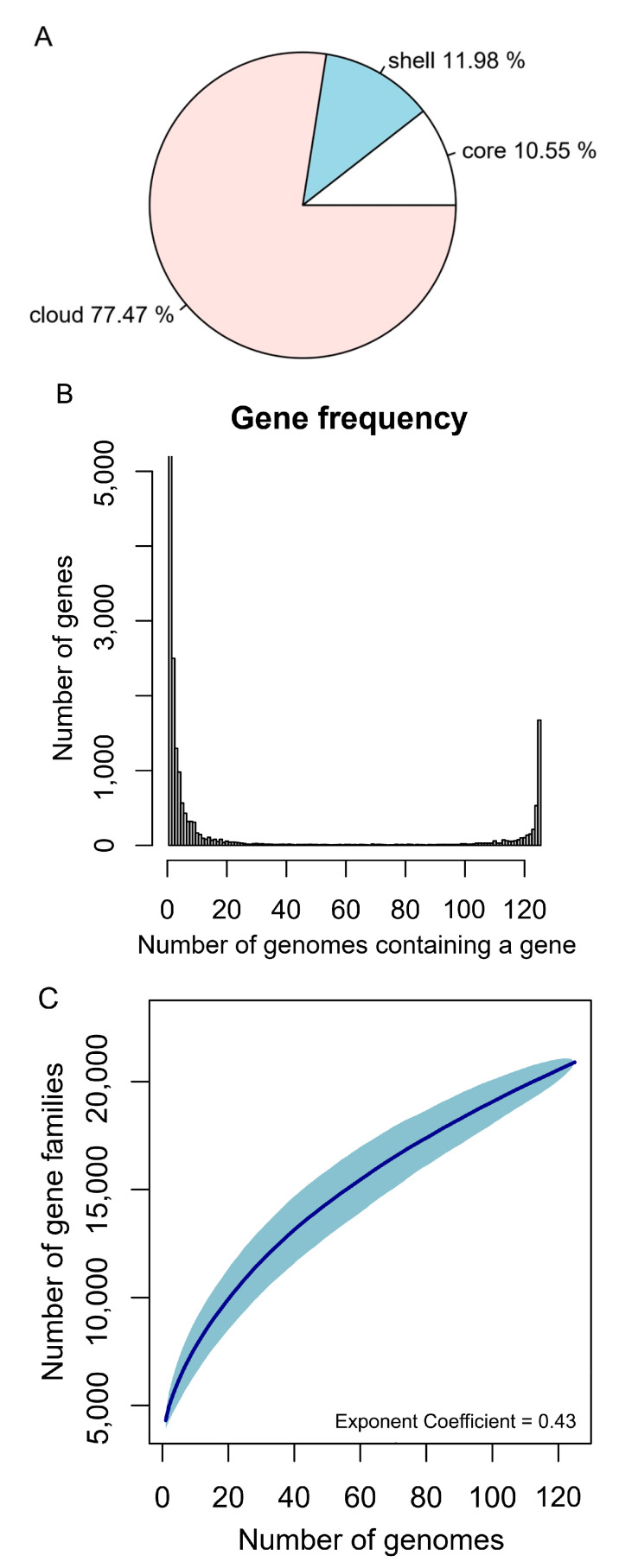
Pangenome dynamics and rarefaction analysis of 125 *S. algae* isolates. (**A**) Pie chart illustrating the pangenome composition of 125 *S. algae* genomes, partitioned into core (10.55%), shell (11.98%), and cloud (77.47%) gene fractions; (**B**) gene frequency distribution showing the number of genes present across the sampled genomes; and (**C**) pangenome rarefaction curve displaying the cumulative increase in gene families as a function of the number of genomes analyzed.

To explore the genomic architecture of *S. algae* VK101 within the species-wide landscape, a comparative pangenome analysis was performed utilizing a dataset of 125 *S. algae* isolates, including *S. algae* VK101 (Supplementary Materials, Table S2; Figure 3). The pangenome was partitioned into three distinct categories based on gene prevalence as follows: core (10.55%), shell (11.98%), and cloud (77.47%) fractions (Figure 3A). Here, we observed significant genetic heterogeneity within the species; approximately 1000 core genes were conserved across all isolates, whereas a vast majority of the total gene content (comprising 2000–5000 genes) was found in a minor subset of *Shewanella* genomes (Figure 3B). The pangenome rarefaction analysis revealed a continuous increase in the cumulative number of gene families relative to the expansion of the genome sampling (Figure 3C). The calculated exponent coefficient of 0.43 from [28] showed that *S. algae* possessed an open pangenome, in which its size could increase indefinitely as new genomes were added.

**Figure 4:**
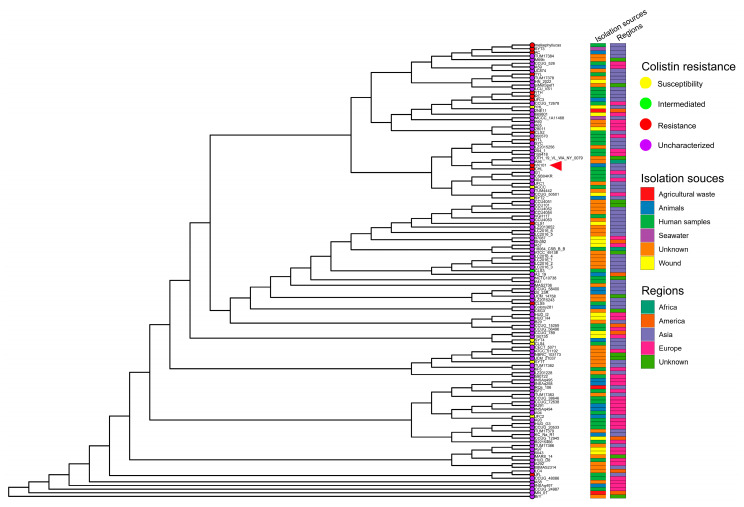
Phylogenetic distribution and ecological origins of the *S. algae* lineage. Maximum-likelihood phylogenetic tree based on the presence/absence of accessory genes across 125 *S. algae* isolates. Color-coded metadata strips indicate colistin-resistance status (red: resistant; yellow: susceptible), isolation source (e.g., human, animal, environmental), and geographical region. The red arrowhead marks the phylogenetic position of *S. algae* VK101.

The phylogenetic reconstruction, derived from the presence–absence matrix of accessory genes, indicated that VK101 was clustered with *S. algae* A56, CHL, and OTH_19_VL_WA_NY_0079 isolates (Figure 4). Interestingly, VK101, despite its environmental isolation from the intestinal microbiome of the black tiger shrimp (*P. monodon*), was clustered closely with those of human clinical isolates. While VK101 exhibited high topological proximity to the colistin-resistant strain CHL, the majority of available public *S. algae* genomes lacked corresponding phenotypic metadata regarding colistin susceptibility, precluding species-wide resistome-phenotype correlations. An average nucleotide identity (ANI) analysis also supported our observation, with all 125 isolates exhibiting 98–99% sequence similarity, thereby confirming high-confidence taxonomic classification within the same species (Supplementary Materials, Figure S1).

### 2.5. Comparative Analysis of Potential Chromosomal Colistin-Resistance Determinants

**Figure 5:**
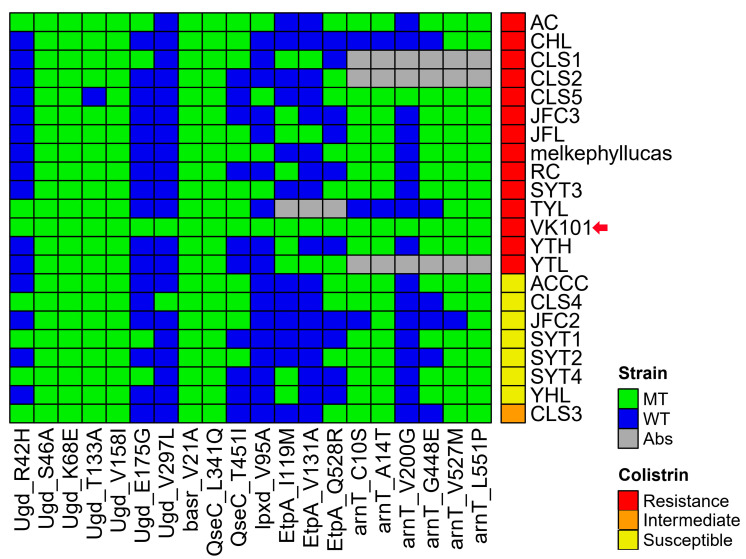
Heatmap of non-synonymous variants in colistin-responsive determinants. Comparative analysis of amino acid substitutions in candidate colistin-resistance genes across 21 *S. algae* isolates. Cells represent mutant (MT, green), wild-type (WT, blue), or absent (Abs, gray) residues compared with the reference strain. The red arrow marks the position of *S. algae* VK101. Phenotypic colistin-resistance status is indicated by the colored sidebar (red: resistant; orange: intermediate; yellow: susceptible).

To elucidate the genetic basis for the high-level colistin resistance observed in *S. algae* VK101, we systematically examined established chromosomal determinants associated with lipid A modification and two-component regulatory systems. We performed a high-resolution comparative analysis of candidate genes—including *ugd*, *qseC*, *lpxD*, *basR*, *eptA*, and *arnT*—across *S. algae* VK101 and a reference dataset of both colistin-resistant and susceptible isolates (Figure 5).

A comparative heatmap analysis revealed that amino acid substitutions in *ugd* and *basR* were nearly ubiquitous across all tested *S. algae* isolates. Importantly, the specific patterns of these substitutions remained conserved across the population and did not significantly differ between colistin-resistant and susceptible strains (Figure 5). While some residues in *qseC*, *lpxD*, *eptA*, and *arnT* remained conserved, the variants that did occur were largely shared across the population regardless of their phenotypic susceptibility.

To determine if these variants exerted functional determinants, we employed a SIFT (sorting intolerant from tolerant) analysis, categorizing scores below 0.05 as deleterious (Table 4). This analysis identified multiple amino acid substitutions predicted to impair protein function. Within the two-component regulatory systems, the sensor kinase, PhoQ harbored a significant R441C mutation (score 0.00), while the phosphoethanolamine transferase (*PmrC*, *eptA*) contained deleterious mutations at positions I119M (score 0.04), V131A (score 0.04), and Q528R (score 0.00). Additional deleterious substitutions were observed in the lipid A biosynthesis protein LpxD (V95A) and the phosphoethanolamine transferases PmrK (C10S, A14T) and PmrX (G448E, V527M). However, the presence of these deleterious variants in both phenotypically resistant and susceptible isolates suggested that these canonical pathways were likely not the primary drivers of the observed 8 μg/mL colistin MIC in the VK101 isolate. This lack of phenotypic–genotypic correlation among traditional determinants would require an approach beyond established models and the utilization of genome-wide association studies (GWAS) to identify genomic loci associated with colistin resistance in the *Shewanella* genus. Such associations provide a foundation for subsequent functional analyses aimed at elucidating novel, non-traditional resistance contributing to trait dissemination.

### 2.6. Association Analysis of Genes and Variants Correlated with Colistin Resistance in S. algae VK101

**Figure 6:**
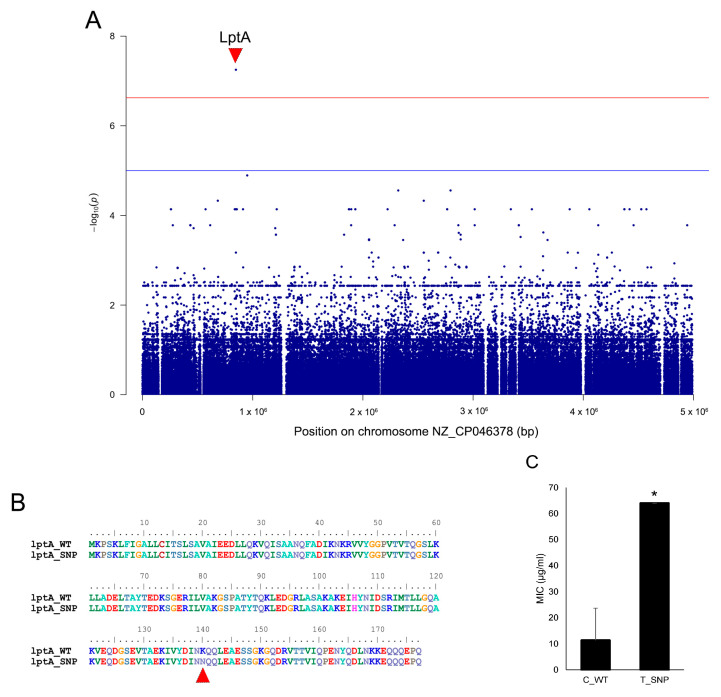
Variant-based association study of colistin-resistance mechanisms. (**A**) Manhattan plot of 210,755 SNPs analyzed for association with colistin resistance. The red triangle identifies a high-confidence SNP in the *lptA* gene exceeding the Bonferroni-adjusted significance threshold (redline, *p*-value = 0.011), while the blue line represents the unadjusted-significance threshold (*p*-value < 0.05), (**B**) sequence alignment of the LptA protein showing the K140N mutation (red triangle), and (**C**) comparison of colistin MIC values between wild-type (C_WT) and mutant (T_SNP) isolates, demonstrating that the K140N variant was related to strains exhibiting high-level resistance. Asterisk (*) indicates statistically significant difference between groups (*p*-value < 0.05).

To identify the novel genetic determinants contributing to colistin resistance in *S. algae*, we performed a GWAS using the presence–absence profiles of the accessory pangenome across the 125-isolate dataset. This analysis identified 29 genes significantly associated with the colistin-resistant phenotype (Supplementary Materials, Table S3). Among these, the top ten candidates including *czcA2* (cobalt–zinc–cadmium resistance), *dmlR* (transcriptional regulator), *ampC* (beta-lactamase), *oprM* (outer membrane protein), and the *pqiA/B* intermembrane transport system, showed 100% sensitivity and 86.67% specificity with an infinite odds ratio, respectively (Table 5). Functional annotation further revealed that these associated determinants were primarily involved in defense mechanisms, transmembrane transport, and cell wall biogenesis (Supplementary Materials, Figure S2). To achieve a higher resolution, we conducted a variant-based association study analyzing 210,755 SNPs across the core genome (Figure 6). This analysis identified only one high-confidence SNP (C > T) that was significantly associated with colistin resistance (Bonferroni-adjusted threshold of *p* = 0.011) (Figure 6A). The SNP (C > T) was found in *lptA,* which encodes for the lipopolysaccharide transport system protein within a mutation from lysine to asparagine at position 140 (Figure 6B). The SNP was found in *lptA* in *S. algae* isolates that survived a very high dose (64 µg/mL) of colistin, while other isolates, including *S. algae* VK101 (which survived at 8 µg/mL), had no changes in *lptA* (Figure 6C).

## 3. Discussion

Here, we characterized the complete genome of the 5.21 Mb genome of *S. algae* VK101, which contained a dominant 4.95 Mb circular chromosome. Our assembly size was consistent with other reported *S. algae* genomes ranging from 4.60 to 5.20 Mb [29,30]. Our pangenomic analysis also showed that *S. algae* possessed an open pangenome, as evidenced by a continuous increase in new gene families and an exponent coefficient of 0.43. This genetic flexibility suggests a high capacity for horizontal gene transfer and adaptation to diverse ecological niches, including aquaculture environments [28]. Furthermore, the phylogenetic analysis revealed that *S. algae* VK101 shared a close evolutionary lineage with colistin-resistant isolates recovered from diverse human and environmental reservoirs. This observation shows the potential for the inter-sectoral transmission of resistant bacteria, thus supporting the necessity of integrated One Health surveillance to monitor the dissemination of such microorganisms across the aquaculture value chain.

Phenotypically, VK101 exhibited its resistance profile to ampicillin and colistin, alongside intermediate susceptibility to imipenem and ciprofloxacin. Mobile colistin-resistance (*mcr*) genes, ranging from variants *mcr-1* to *mcr-10*, are globally recognized as primary drivers of colistin resistance [31,32]. Interestingly, despite its colistin-resistant phenotype, no *mcr* homologs were detected within the genome of *S. algae* VK101. Our findings were consistent with a previous study of genomic evaluations of *S. algae* isolates [30] and the studies of other colistin-resistant bacterium, *Acinetobacter baumannii*, which similarly lacked mobile *mcr* elements [33,34]. This genotype–phenotype gap suggests that colistin resistance in *S. algae* could be predominantly mediated by chromosomal mechanisms rather than horizonal gene transfer. Our SIFT-based functional analysis supported this hypothesis, identifying several deleterious mutations (score ≤ 0.05) in chromosomal colistin-responsive determinants. These included R441C substitution in the sensor kinase PhoQ and multiple substitutions within the *pmrCAB* operon, specifically in the phosphoethanolamine transferase PmrC/eptA (I119M, V131A, and Q528R). Alterations in the *pmrCAB* operon has been well characterized as one of the key mechanisms for colistin resistance in clinical pathogens such as *A. baumannii* [35]. Furthermore, significant functional disruptions were predicted for PmrK/arnT, which harbored six deleterious mutations (C10S, A14T, V200G, G448E, V527M, and L551P), for instance, the enzymatic modification of lipid A or the structural disruption of lipopolysaccharide (LPS) biosynthesis [33,36,37]. The addition of phosphoethanolamine to lipid A, which is regulated by phosphoethanolamine transferases (*pmr*) and lipid A biosynthesis (*lpx*), reduces the negative charge of the bacterial outer membrane, thereby reducing its binding affinity for cationic polymyxin groups including colistin [36]. Within the *Shewanella*, previous studies have reported specific combinatorial mutations in the *pmrB*, *pmrE*, and *pmrH* genes as primary drivers of colistin resistance [30]. Collectively, these modifications likely facilitate lipid A modification, providing a mechanistic insight into the resistance observed in *S. algae* VK101. Beyond colistin, chromosomal analysis also identified determinants for other antibiotic classes including a perfect identity match (100%) for the QnrA3 protein, explaining the intermediate susceptibility to fluoroquinolones [38]. The presence of the OXA-964 beta-lactamase (98.96%) and RND-family efflux pumps like *rsmA* further defined the isolate’s multidrug-resistant profile [39].

While our GWAS identified significant associations between the colistin-resistant phenotype and several genes (*czcA*, *ampC*, *oprM*, and *pqiAB*), these will further require functional validation to understand biological causality. Given the lack of existing evidence connecting these genes to primary colistin-resistance mechanisms, their high statistical association could likely be driven by a combination of genetic collinearity (co-selection) and indirectly by membrane stability and efflux systems. For instance, *czcCBA*, a tripartite RND efflux pump system that mediates resistance to heavy metals, also contributes to carbapenem resistance by suppressing *oprD* porin in *P. aeruginosa* [40]. Furthermore, the deletion of *czcA* results in reducing tetracycline resistance, while overexpression of *crdAB*-*czcBA* through copper supplementation decreases tetracycline efflux in *Helicobacter pylori* [38]. Additionally, multidrug-resistant *A. baumannii* AC30 and R14, which exhibit resistance to carbapenems and colistin, shows the presence of *czcCBA* [41]. This suggests that *czcA* may be co-selected in multidrug-resistant genetic backgrounds or implicated in indirect colistin tolerance via secondary efflux transporters. Similarly, *ampC* (a beta-lactamase) typically confers beta-lactam resistance by hydrolyzing the beta-lactam ring [42], frequently found co-expressed with ESBL and MBL genes in colistin-resistant bacteria such as *K. pneumoniae*, *E. coli*, and *P. aeruginosa* [43,44]. This suggests a co-selection environment rather than a direct colistin degrading mechanism. Moreover, the efflux pumps via *oprM*, an outer membrane channel protein that is responsible for the direct export of antibiotics from the cells [45], can impart resistance to several drugs, including beta-lactams, fluroquinolones, and aminoglycosides in *P. aeruginosa* [46]. Notably, the efflux pump *mexXY-oprM* is implicated in the PA4773-PA4774-PA4775 pathway and *ara4N*-based modification of LPS during colistin resistance in *P. aeruginosa* [46]. Therefore, colistin resistance in *S. algae*, including our isolate, *S. algae* VK101, might be partially supported by these indirect efflux mechanisms. Finally, *pqiABC* regulates lipid transport and membrane integrity in association with the *yebST* operon and Mla pathway [47,48]. While direct evidence is still lacking, enhancing the Mla pathway (e.g., through an *mlaF* mutation) could drive colistin resistance in *Aeromonas hydrophila* [49]. Consequently, *pqiABC* might indirectly influence colistin resistance in *S. algae* by modulating this shared Mla transport pathway.

The phenotypic variation in colistin susceptibility within the *S. algae* population suggests complex regulatory components. While *S. algae* VK101 exhibited a moderate resistance phenotype (8 µg/mL), this was notably lower than the high-level resistance (64 µg/mL) recently reported in other *S. algae* isolates [30]. This suggests that the moderate resistance in *S. algae* VK101 was likely driven by the intrinsic chromosomal pathways, potentially involving lipid A modifications. It is worth noting that a definitive classification of these resistance levels remains ambiguous due to the absence of established MIC criteria for *Shewanella* spp. To elucidate the broader species-wide mechanisms driving high-level resistance, our GWAS identified a non-synonymous mutation (K140N) in the *lptA* gene significantly associated with an MIC of 64 µg/mL) [30]. The LptA protein is a central component of the trans-envelope machinery [50]. We hypothesize that substituting a positively charged lysine with a neutral asparagine (K140N) at the LptA C- terminus destabilizes its interaction with the N-terminal domain of LptD [51,52]. This impaired assembly likely decreases in LPS transport and depletes surface LPS molecules, thereby limiting the binding targets available for colistin [53]. Future structural modeling and functional studies such as assays measuring LPS levels, transport efficiency, and direct colistin binding will be crucial to further validate and understand impact of this mutation on antibiotic resistance.

Viewed through a One Health framework, characterizing multidrug-resistant *S. algae* VK101 from wild-caught *P. monodon* broodstock demonstrated environmental AMR reservoirs as crucial components of the aquaculture value chain. Our findings showed that colistin resistance could be driven by complex chromosomal modifications, suggesting that traditional molecular screening for mobile *mcr* genes may no longer be sufficient for comprehensive risk assessment. To mitigate the dissemination of these non-canonical determinants, it is imperative to move beyond static resistance databases toward integrated genomic surveillance that prioritizes functional variant analysis. Establishing these baseline genomic signatures will provide a foundation for the precision biosecurity protocols and sustainable management strategies necessary to safeguard global food security and public health.

## 4. Materials and Methods

### 4.1. Microbial Culture Conditions and DNA Preparation

Bacterial species isolated from the intestines of black tiger shrimp (*P. monodon*) were subjected to whole-genome analysis. The bacterial isolate was streaked onto Luria–Bertani (LB) agar and incubated at 30 °C for 16 h. Twenty single bacterial colonies were used for DNA extraction using the ZymoBIOMICS DNA Miniprep kit (Zymo Research, Irvine, CA, USA) according to the manufacturer’s protocol. The DNA sample was cleaned up using AMPure PB bead (Pacific Biosciences, Menlo Park, CA, USA). The extracted DNA was quantified by NanoDrop 8000 spectrophotometer (Thermo Fisher Scientific, Waltham, MA, USA) and Qubit dsDNA BR Assay kit (Invitrogen, Carlsbad, CA, USA) using a Qubit 4.0 fluorometer (Invitrogen, USA). The DNA quality and integrity were visualized by UV light after electrophoresis in 0.8% agarose gel.

### 4.2. Library Preparation and Bacterial Genome Sequencing

Whole-genome sequencing was performed using a hybrid approach combining Illumina short-read and Oxford Nanopore Technologies (ONT) long-read platforms. For short-read sequencing, a paired-end (2 × 150 bp) library was prepared using the NEBNext^®^ Ultra™ II DNA Library Prep Kit (New England Biolabs, Ipswich, MA, USA) and sequenced on the Illumina NovaSeq platform (Illumina, San Diego, CA, USA). For long-read sequencing, a library was constructed using the Rapid Barcoding Kit (RBK004; Oxford Nanopore Technologies, Oxford, UK). Sequencing was performed on a MinION Mk1C device equipped with an R10.3 flow cell for 48 h.

Illumina sequencing adapters were trimmed using Fastp v0.19.5 with default parameters, and the quality of clean reads was assessed using FastQC v0.11.9. ONT raw signals were base called using Guppy v5.0.16 with the super-accurate model (dna_r10.3_450bps_sup.cfg). Adapters were removed using Porechop v0.2.4. Long-read quality was assessed using NanoPlot v1.28.1, and reads were filtered using NanoFilt v2.5.0 [54] to retain reads of > 1000 bp with a mean quality score (Q-score) of > 8. A de novo hybrid assembly was performed using Unicycler v0.4.4 [55]. The quality of the final assembly was evaluated using QUAST v5.0.2 [56] and BUSCO v5.8.3 [57]. Genome annotation was performed using the NCBI Prokaryotic Genome Annotation Pipeline (PGAP) v4.11 [58].

### 4.3. Genetic Analysis of Chromosomal Genes Associated with Colistin Resistance

Genomic sequences of isolate VK101 were screened for candidate genes previously implicated in colistin resistance, including *lpxA*, *lpxC*, *lpxD*, *phoP*, *phoQ*, *pmrA*, *pmrB*, *pmrC*, *pmrH*, *pmrK*, and *pmrX*). Protein-coding sequences from those genes were extracted and aligned against the sequence of *Shewanella algae* CECT-5071 reference strain (GenBank accession CP068230.1) using MUSCLE v5.1 [59], and non-synonymous mutations were identified by pairwise comparison. The functional impact of each amino acid substitution was predicted using the sorting intolerant from tolerant (SIFT) algorithm [60] using the SIFT web server (https://sift.bii.a-star.edu.sg/, accessed on 10 October 2025) with default parameters. SIFT calculates the probability that an amino acid substitution at a given position is tolerated based on sequence homology and the frequency of amino acids observed at that position. Substitutions with normalized values ≤ 0.05 were classified as deleterious, indicating a high likelihood of functional disruption and potential association with colistin resistance.

### 4.4. MIC Method

The antimicrobial susceptibility of VK101 was evaluated using the broth microdilution method with commercially prepared, dehydrated 96-well microtiter plates, THAN2F (Sensititre, Trek Diagnostic Systems Ltd., West Sussex, UK). A total of 21 antibiotics from different drug classes and mechanisms of action were included in this study (Table 2). MIC tests were performed according to the manufacturer’s instruction with minor modifications. Briefly, VK101 were streaked onto sheep blood agar and incubated for 24 h at 37 °C. The selected colonies were suspended in Sensititre cation-adjusted Mueller–Hinton broth with TES buffer (CAMHBT) and adjusted to be a 0.5 McFarland standard. Subsequently, a 30 µL aliquot of the suspension was transferred into a tube of CAMHBT to obtain an inoculum density of 5 × 10^5^ CFU/mL. The THAN2F antibiotic panels were reconstituted by adding 50 µL per well, sealed with an adhesive seal and incubated at 35 ± 2 °C in Sensititre ARIS^TM^ 2X system for 20–24 h. The MIC value, defined as the lowest drug concentration inhibiting visible bacterial growth, was determined automatically by the Sensititre ARIS^TM^ 2X and verified visually using a manual viewbox, according to the instructions in the Sensititre SWIN software v 3.4. *Escherichia coli* ATCC 25922 was used as an antimicrobial-susceptible control strain, and MICs were within the accepted quality control ranges. As there were no established MIC breakpoints specifically for *Shewanella* spp., the results of susceptible (S), intermediate (I), and drug resistance (R) were interpreted according to the *Enterobacterales* standards of Clinical and Laboratory Standards Institute [27]. The application of *Enterobacterales* criteria is a widely adopted and standardized approach for interpreting susceptibility in related non-fermenting Gram-negative bacilli including *Shewanella* when species-specific clinical breakpoints are unavailable [61].

### 4.5. Comparative Genomics of S. algae VK101 and In Silico Antibiotic Resistant Gene Identification

The genomes of *S. algae* VK101 and other *S. algae* were compared. The 125 reported *S. algae* genomes were retrieved from the NCBI database. The list of *S. algae* used in this study was shown in the Supplementary Materials, Table S2. The genomes of *S. algae* were annotated using Prokka v. 1.14.6 [62] with the *S. algae* database. Then, the generic feature format (gff) files obtained from the annotation results of these *S. algae* were used for comparative analysis using Roary v. 3.13.0 [63]. The output of absent and present genes was used to construct a phylogenetic tree using FastTree v.2.1 [64]. In addition, average nucleotide identity (ANI) was analyzed among these *S. algae*, including VK101, by FastANI v. 1.33 [65].

According to the examination of colistin-resistant ability of *S. algae* isolates [30], we retrieved these genomes, including 7 susceptible and 14 resistant ones, together with our *S. algae* VK101 for genes and variant analyses (Supplementary Materials, Table S1). The reported colistin MIC values for *S. algae* isolates were utilized as phenotypic data to analyze gene absence/presence associations using Scoary v. 1.6.16 [66], applying a naive *p*-value threshold of <0.05 and an odds ratio of >10. The related colistin-resistant genes were functionally annotated by emapper-eggNOG via the eggNOG database [67] as well as gene ontology (GO), COG, and the KEGG pathway. In addition, these genomes were compared with the reference genome of *S. algae* RQs-106 (RefSeq Accession No. GCF_009730655.1) using ParSNP v. 1.7.4 [68] with default parameters. The SNPs were further filtered for missing genotype rates of ≤5% and minimum allele frequencies of ≤0.01 using PLINK v.1.9 [69]. The SNPs were then analyzed for association with the colistin resistance using PLINK v. 1.9 using Fisher’s test and Bonferroni’s *p*-value adjustment. The genome of *S. algae* VK101 was analyzed for antibiotic resistant genes using resistant gene identifier v. 6.0.5 against CARD v. 4.0.1 [70].

## 5. Conclusions

In this study, we successfully resolved the 5.21 Mb complete circular genome of *Shewanella algae* VK101, isolated from the intestinal microbiome of wild-caught *Penaeus monodon* broodstock. Our multi-platform sequencing approach confirmed that this isolate contained a diverse resistome, including a perfect hit for the fluoroquinolone resistance gene *qnrA3*, and exhibited phenotypic resistance to both ampicillin and colistin. Interestingly, the absence of mobile *mcr* genes suggested that colistin resistance in this environmental isolate was mediated by chromosomal mechanisms. Our GWAS identified a novel K140N mutation in the lipopolysaccharide transport protein gene (*lptA*) associated with high-level colistin resistance. While this mutation was absent in VK101, this study showed a diverse landscape of colistin resistance in aquaculture reservoirs, ranging from moderate chromosomal-mediated resistance to high-level structural modifications. Under a One Health framework, our results support the urgent need for integrated genomic surveillance in aquaculture to monitor the dissemination of non-canonical resistance determinants through the food production chain to safeguard public health.

## Figures and Tables

**Table 1:** Genomic features and assembly metrics for the complete *Shewanella algae* VK101 genome. The genome was reconstructed using a hybrid assembly strategy integrating Illumina short-read and Oxford Nanopore long-read data. Statistics include total genome size, assembly contiguity (N50), and functional annotation metrics including coding sequences (CDS), transfer RNAs (tRNAs), and ribosomal RNAs (rRNAs).

	VK101
Genome size (Four complete circular contigs, Mb)	5.21
Genome size (Domain chromosome, Mb)	4.95
Number of reads (Short reads)	8,305,448
Number of reads (Long reads)	305,874
Number of assembled contigs	4
N50 (bp)	4,951,710
Genome size (bp)	5,218,190
GC content (%)	52.6
Number of CDSs	4620.0
Coding Ratio (%)	87.2
Number of rRNAs	25.0
Number of tRNAs	103.0

**Table 2:** Phenotypic antimicrobial susceptibility profile of *Shewanella algae* VK101. Minimum inhibitory concentration (MIC) values for 21 antimicrobial agents across six functional categories. Interpretations of susceptibility (S), intermediate (I), and resistance (R) were determined based on Clinical and Laboratory Standards Institute (CLSI) M100 standards [27] for *Enterobacterales*.

Antimicrobial Category	Antimicrobial Agent	THAN2F Plate (µg/mL)	MIC Breakpoints (µg/mL)			*S. algae* VK101	
			S	I	R	MIC (µg/mL)	Interpretation
Cell wall synthesis inhibitor							
Beta-lactams/	Ampicillin	8–16	≤8	16	≥32	>16	R
Beta-lactamase inhibitors	Ampicillin/Sulbactam	4/2–16/8	≤8/4	16/8	≥32/16	8/4	S
	Amoxicillin/Clavulanic Acid	4/2–16/8	≤8/4	16/8	≥32/16	16/8	I
	Piperacillin/Tazobactam	8/4–64/4	≤8/4	16/4	≥32/4	≤8/4	S
Cephalosporins	Cefoxitin	4–16	≤8	16	≥32	8	S
	Cefuroxime (sodium)	8–16	≤8	16	≥32	≤8	S
	Cefotaxime	1–32	≤1	2	≥4	≤1	S
	Ceftazidime	1–32	≤4	8	≥16	≤1	S
	Ceftriaxone	0.5–32	≤1	2	≥4	≤0.5	S
	Cefepime	1–32	≤2	4–8	≥16	≤1	S
Carbapenems	Imipenem	0.5–16	≤1	2	≥4	2	I
	Meropenem	0.5–16	≤1	2	≥4	≤0.5	S
	Ertapenem	0.5–4	≤0.5	1	≥2	≤0.5	S
	Doripenem	0.5–16	≤1	2	≥4	≤0.5	S
DNA replication inhibitor							
Fluoroquinolones	Ciprofloxacin	0.06–2	≤0.25	0.5	≥1	0.5	I
	Levofloxacin	0.06–8	≤0.5	1	≥2	0.25	S
Protein synthesis inhibitor							
Aminoglycosides	Amikacin	8–32	≤16	32	≥64	≤8	S
	Gentamicin	2–8	≤4	8	≥16	≤2	S
	Netilmicin	8–16	≤8	16	≥32	≤8	S
Outer cell membrane disruptor							
Polymyxins	Colistin	1–8	-	≤2	≥4	8	R
Folate synthesis inhibitor							
Sulfonamides	Trimethoprim/ Sulfamethoxazole	1/19–4/76	≤2/38	-	≥4/76	≤1/19	S

S, Susceptibility; I, Intermediate; R, Resistance.

**Table 3:** Genomic identification of putative antimicrobial resistance genes. Percent identity hits for antimicrobial resistance genes (ARGs) identified in the *Shewanella algae* VK101 genome using the Comprehensive Antibiotic Resistance Database (CARD).

List of ARGs	No. of Hit	AMR Gene Family	Drug Class	Antibiotics	Best Identities (%)
*qnrA3*	1	quinolone resistance protein	fluoroquinolone antibiotic	ciprofloxacin; levofloxacin; moxifloxacin; gatifloxacin; nalidixic acid; norfloxacin; sparfloxacin	100
*OXA-964*	1	OXA beta-lactamase; OXA-55-like beta-lactamase	carbapenem; penicillin beta-lactam	oxacillin	98.96
*Escherichia coli* EF-Tu mutants conferring resistance to Pulvomycin	2	elfamycin resistant EF-Tu	elfamycin antibiotic	pulvomycin	88.30
*rsmA*	1	resistance-nodulation-cell division (RND) antibiotic efflux pump	fluoroquinolone antibiotic; diaminopyrimidine antibiotic; phenicol antibiotic	trimethoprim; chloramphenicol	86.67

**Table 4:** Sorting intolerant from tolerant (SIFT)-based prediction of functional impacts of chromosomal mutations in colistin-resistance determinants. Amino acid substitutions were identified by comparing *S. algae* VK101 against the *S. algae* CECT-5071 reference strain. SIFT scores reflect the predicted tolerance of each substitution based on sequence homology. Shaded cells (red) indicate deleterious mutations (SIFT score ≤ 0.05).

Determinants	Mutations	SIFT Score
LpxA	WT	
LpxC	L270Q	1
LpxD	V95A	0.1
PhoP	Q65R	1
PhoQ	K290R	0.2
	K335E	0.64
	D436A	0.12
	R441C	0
PmrC (*eptA*)	I119M	0.04
	V131A	0.04
	I136T	0.16
	I143M	0.19
	I158V	0.74
	E212K	1
	N287S	0.68
	H307N	0.16
	Q528R	0
PmrA (*basR*, *qseB*)	V21A	0.44
PmrB (*basS*, *qseC*)	L341Q	0.62
	T451I	0.21
PmrH (*arnB*)	A307S	0.56
	S309A	0.08
PmrF (*arnC*)	WT	
PmrK (*arnT*)	C10S	0
	A14T	0.01
	R15K	0.31
	V200G	0
	F334L	1
	R420K	0.3
	G448E	0.01
	V527M	0
	L551P	0
PmrE (*ugd*)	R42H	0.13
	S46A	0.8
	K68E	1
	T133A	0.52
	V158I	0.4
	E175G	0.55
	V297L	0.36

**Table 5:** Top-ranked genes associated with high-dose colistin resistance in *S. algae*. Genomic association analysis identifying the top 10 genes significantly correlated with survival in high concentrations of colistin among 125 *S. algae* isolates. Statistics include sensitivity, specificity, and the odds ratio (Inf = infinite correlation).

Gene	Annotation	Sensitivity	Specificity	Odds Ratio	Naïve *p*-Value
*czcA2*	Cobalt–zinc–cadmium resistance protein	100	86.66667	Inf	0.000211
*dmlR_1*	HTH-type transcriptional regulator	100	86.66667	Inf	0.000211
*ampC*	Beta-lactamase	100	86.66667	Inf	0.000211
*oprM_2*	Outer membrane protein	100	86.66667	Inf	0.000211
*pqiA*	Intermembrane transport protein	100	86.66667	Inf	0.000211
*pqiB*	Intermembrane transport protein	100	86.66667	Inf	0.000211
*BedF_1*	Efflux pump periplasmic linker	100	86.66667	Inf	0.000211
*DsbD_1*	Thiol:disulfide interchange protein	100	86.66667	Inf	0.000211
*cobQ*	Cobyric acid synthase	85.71	86.67	39	0.002345
*hcaR*	Hca operon transcriptional activator	85.71	80	24	0.006614

## Data Availability

The sequencing data of *Shewanella algae* VK101 has been deposited in the Bioproject database under accession number PRJNA1285665.

## Supplementary Materials

The following supporting information can be downloaded at https://www.mdpi.com/article/10.3390/antibiotics15040405/s1.; Figure S1. Average nucleotide identity (ANI) heatmap analysis of *Shewanella algae* genomes; Figure S2. Functional characterization of genes associated with colistin resistance. (A) Distribution of the 29 genes significantly associated with colistin resistance by functional role, (B) COG classification of these associated determinants, and (C) KEGG pathway enrichment; Table S1. Descriptive information of the identified ARGs in *S. algae* VK101 genome; Table S2. Metadata information of *S. algae* genome used in this study; Table S3. Statistical analysis of gene associated with colistin resistance trait; Table S4. Characterization of colistin and polymyxin resistance determinants and mutational tolerance. Genomic profile of putative colistin and polymyxin resistance genes in *Shewanella algae* VK101, categorized by functional role.
