# Supplementary material for: Genomic and GWAS-Based Insights into Antimicrobial Resistance in Shewanella algae Isolated from Penaeus monodon
Source: Antibiotics (Basel). 2026 Apr 16;15(4):405. doi: 10.3390/antibiotics15040405 (PMC13113632; doi:10.3390/antibiotics15040405)

Supplemental Figure S2. Functional characterization of genes associated with colistin resistance. (A) Distribution of the 29 genes significantly associated with colistin resistance by functional role, **(B)** COG classification of these associated determinants, and **(C)** KEGG pathway enrichment.

A

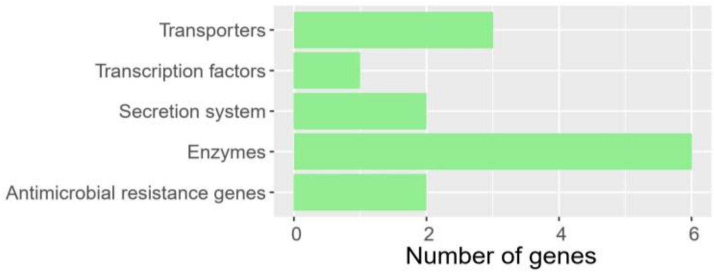

B

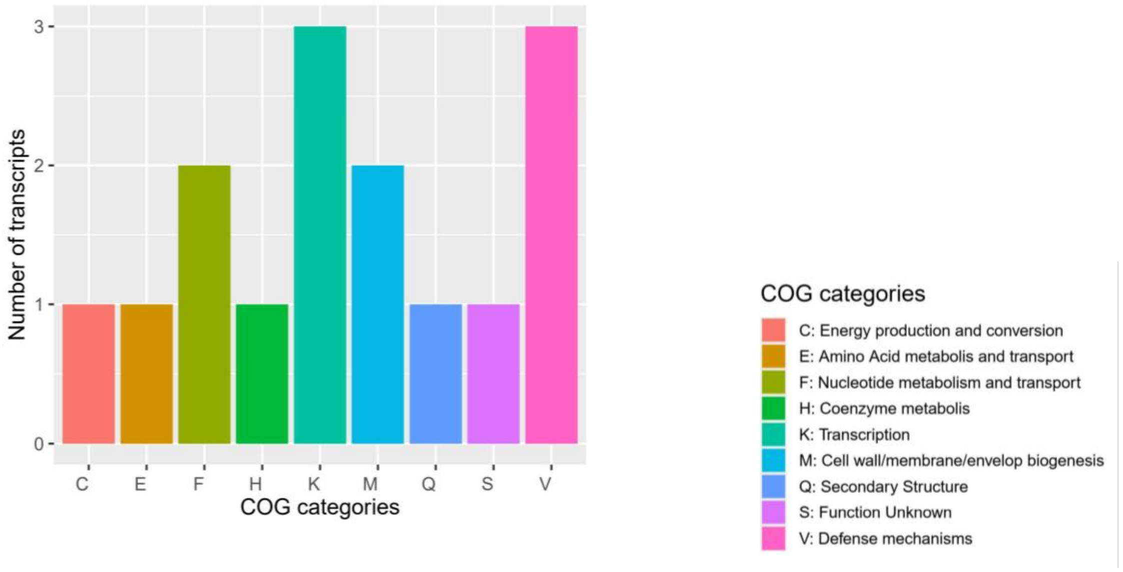

C

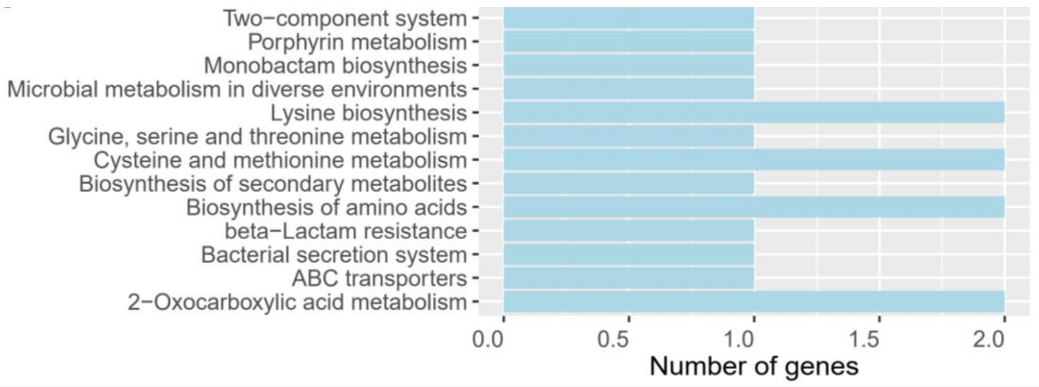

Supplement: Supplementary file 1 [file antibiotics-15-00405-s001.zip › Fig S2 Functional annotation of genes associated with colistin resistance.pdf]
