# Supplementary material for: Genomic and GWAS-Based Insights into Antimicrobial Resistance in Shewanella algae Isolated from Penaeus monodon
Source: Antibiotics (Basel). 2026 Apr 16;15(4):405. doi: 10.3390/antibiotics15040405 (PMC13113632; doi:10.3390/antibiotics15040405)
Supplement: Supplementary file 1 [file antibiotics-15-00405-s001.zip › Table S4 The related colistin A and polymyxin B resistant genes in VK101.pdf]

Table S4. Characterization of colistin and polymyxin resistance determinants and mutational tolerance. Genomic profile of putative colistin and polymyxin resistance genes in *Shewanella algae* VK101, categorized by functional role.

**Gene function**	**List of related ARGs**	**Mutational tolerance (SIFT)**
Phosphoethanolamine transferase (*pmr*)	Ugd (*pmrE*)	-
	EptA (*pmrC*)	I119M, V131A, Q523R
	ArnT (*pmrK*)	C10S, A14T, V200G, G448E, V527M, L551P
	QseC (*pmrA*)	-
	QseB (*pmrB*)	-
	ArnB (*pmrH*)	-
Two-component system	PhoP	-
Lipid A biosynthesis	LpxA	-
	LpxC	-
	LpxD	-
	LpxM	-
	LpxL	-
	LpxH	-
