# Supplementary figures and images for: Genomic and GWAS-Based Insights into Antimicrobial Resistance in Shewanella algae Isolated from Penaeus monodon
Source: Antibiotics (Basel). 2026 Apr 16;15(4):405. doi: 10.3390/antibiotics15040405 (PMC13113632; doi:10.3390/antibiotics15040405)

Supplemental Figure S1

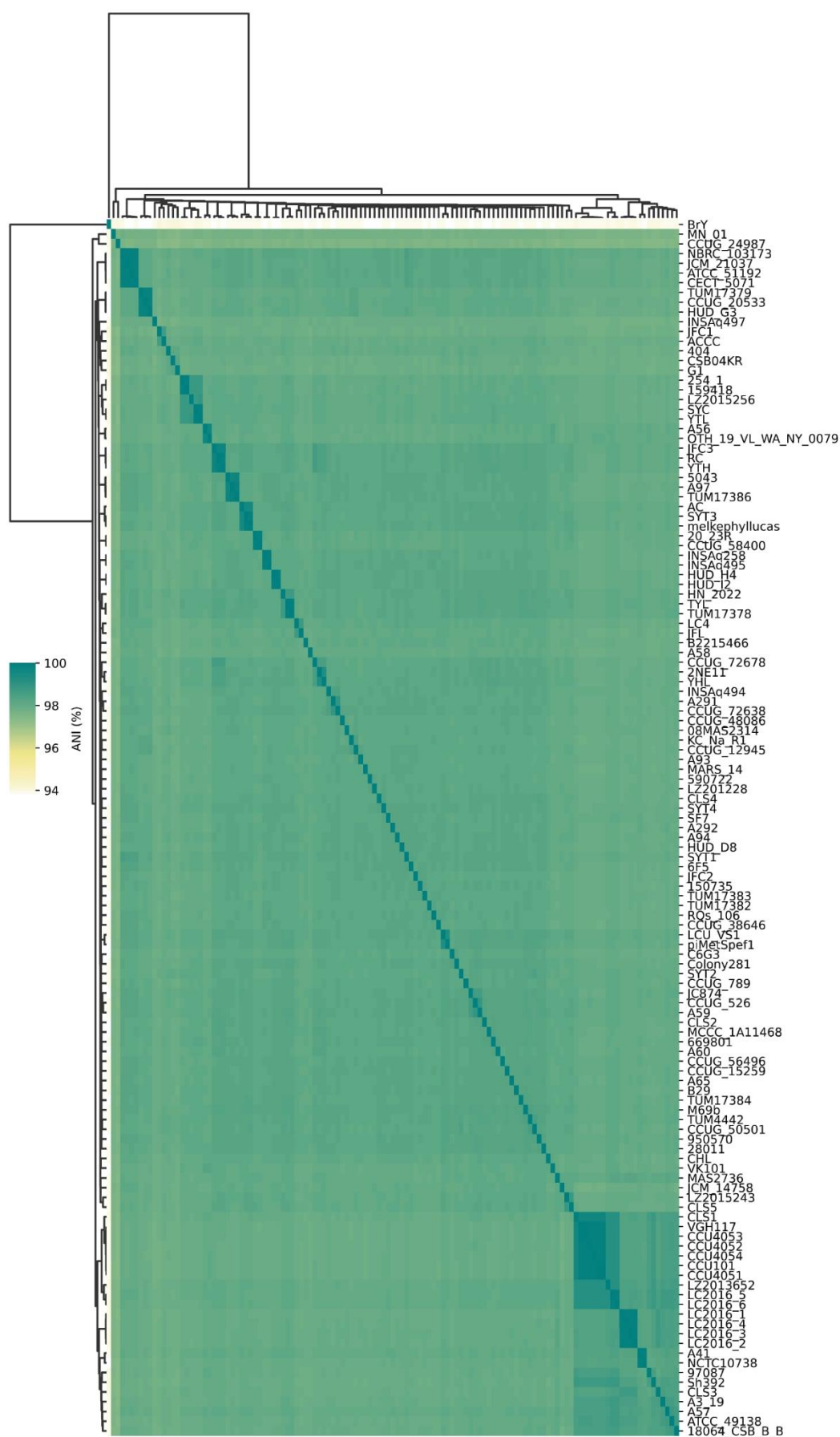

Supplement: Supplementary file 1 [file antibiotics-15-00405-s001.zip › Fig S1 ANI edited.pdf]
